# Prenatal intraventricular hemorrhage in a term infant with congenital CMV infection

**DOI:** 10.11604/pamj.2015.22.244.8156

**Published:** 2015-11-13

**Authors:** Imene Dahmane Ayadi, Emira Ben Hamida

**Affiliations:** 1 Department of Neonatology, Charles Nicole Hospital, Tunis El Manar University, Tunis, Tunisia

**Keywords:** Congenital, cytomegalovirus, intraventricular hemorrhage, newborn

## Image in medicine

Intraventricular hemorrhage (IVH) occurs rarely in term infant, since subependymal area is a transient structure in fetal life. IVH in term infant indicates generally that it happened prenatally. Congenital cytomegalovirus (CMV) infection is frequent, occurring in 1% of live births. It is a severe infection leading to developmental defects, especially sensorineural deafness. The diagnosis of congenital CMV infection is rarely evoked in term eutrophic newborn. We report a term male neonate born to a 32-year-old mother gravida 2, para 2. Pregnancy was uneventful. Ultrasound follow-up was unmarked. The newborn was eutrophic, birth weight was 3400g, length was 49cm, and head circumference 33cm. Neonatal examination showed no anomalies. On the first day of life, blood cells count performed for suspected materno-fetal infection discovered fortuitous thrombocytopenia at 30 x 109/L. Within the second day of life, platelet level dropped to 20 x 109/L. Management of thrombocytopenia included multiple platelet transfusion. Cranial ultrasound on day one of life showed bilateral subependymal hemorrhage with cysts (A) and hydrocephaly (B), signs of prenatal occurrence. Platelet phenotypage rules out the diagnosis of allo-immune thrombocytopenia. Laboratory testing for rubella was negative. Blood PCR CMV was positive. Liver function tests noted the absence of hepatic cytolysis and cholestasis. The newborn received intravenous Ganciclovir^®^ therapy. Hearing screening before discharge was negative. Bilateral deafness was diagnosed at 3 months of life.

**Figure 1:**
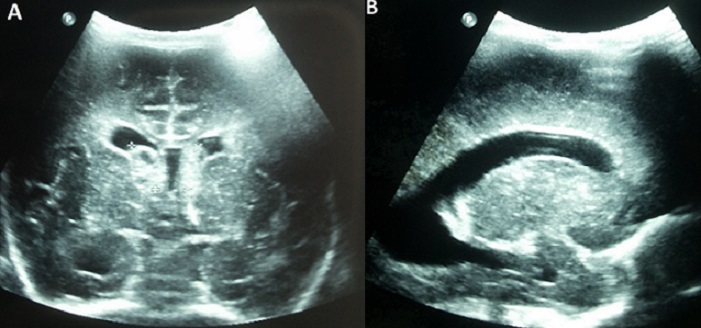
(A) bilateral subependymal hemorrhage; (B) hydrocephaly

